# The paradox of the first cycle of chemotherapy—transient improvement of contractility and diastolic function after the first cycle of anthracycline-based chemotherapy: a prospective clinical trial

**DOI:** 10.18632/oncotarget.21279

**Published:** 2017-09-27

**Authors:** Paweł Stachowiak, Andrzej Wojtarowicz, Marta Milchert-Leszczyńska, Krzysztof Safranow, Michał Falco, Robert Kaliszczak, Zdzisława Kornacewicz-Jach

**Affiliations:** ^1^ Department of Cardiology, Pomeranian Medical University, Szczecin, Poland; ^2^ Department of Radiotherapy, West Pomeranian Oncology Center, Pomeranian Medical University, Szczecin, Poland; ^3^ Department of Biochemistry and Medical Chemistry, Pomeranian Medical University, Szczecin, Poland

**Keywords:** anthracycline, breast cancer, cardiotoxicity, diastolic function, speckle tracking

## Abstract

**Aims:**

Breast cancer is the most common cancer among women, and anthracyclines are the most commonly administered drugs for these patients. Cardiotoxicity is one of the complications, which limits the success of this therapy. Very few studies have evaluated anthracycline toxicities within the first few hours after the first infusion, and the majority of published studies were performed in animal models. The present study aimed to evaluate changes in echocardiographic parameters in women with breast cancer 24 hours after receiving the first dose of an anthracycline.

**Materials and Methods and Results:**

The present study included 75 chemotherapy-naive female patients without heart failure, who were diagnosed with breast cancer and were scheduled to undergo anthracycline-based chemotherapy (epirubicin and doxorubicin). During their visits to the Heart Center, the patients underwent detail echocardiographic examination, including assessment of systolic and diastolic function and longitudinal strain. There were no differences in baseline echocardiographic parameters between patients with and those without cardiotoxicity. Cardiotoxicity was observed during follow-up in 14 patients (18.7%). Improvements in left ventricular ejection fraction and global longitudinal strain were observed at 24 hours after administration of the cytotoxic agent in the subgroup of patients without further cardiotoxicity. The changes were transient and the assessment of left ventricular ejection fraction after completion of chemotherapy revealed similar values to those before the treatment.

**Conclusions:**

The findings of our study suggest that transient improvement in contractility and systolic and diastolic function might occur 24 hours after anthracycline administration, especially in patients who do not develop cardiotoxicity.

## INTRODUCTION

Breast cancer is a social problem, and its management involves surgery, chemotherapy, and radiotherapy. However, many drugs used in chemotherapy for this malignancy are cardiotoxic. Anthracyclines have been reported to be the most commonly used antineoplastic agents [1].

As the cardiotoxicity of chemotherapy drugs is dependent on multiple factors [2] and the time to cardiotoxicity onset varies, close cardiovascular monitoring during cancer treatment is required [3]. Echocardiography is the principal modality to establish the presence of drug-induced cardiotoxicity and to monitor this cardiotoxicity. It is a non-invasive, relatively easily accessible, and inexpensive examination that enables the rapid evaluation of left ventricular ejection fraction (LVEF) [4]. The past decade has seen considerable progress in the field of echocardiographic techniques, and although many parameters can currently be used to describe systolic function, the assessment of ejection fraction remains extremely important in the evaluation of cardiotoxicity [5, 6].

Previous clinical studies focused on the cardiovascular effects of chemotherapy after its completion, when the toxic effects are most evident [7–9]. Very few studies have evaluated anthracycline toxicities within the first few hours after the first infusion, and the majority of published studies were performed in animal models.

The present study aimed to evaluate the changes in echocardiographic and laboratory parameters in women with breast cancer 24 hours after receiving the first dose of an anthracycline.

## RESULTS

The study included 75 female patients with breast cancer. Each patient received anthracycline therapy. In addition, the patients received cyclophosphamide (82.7%), 5-fluorouracil (17.3%), docetaxel (25.3%), and trastuzumab (21.6%), with the latter being started after completion of chemotherapy and radiotherapy. The two anthracyclines used were doxorubicin (84.0% of the patients) and epirubicin (16.0% of the patients). The mean anthracycline dose, expressed as doxorubicin dose, was 267.7 ± 52 mg/m^2^.

Among the 75 patients who participated in the study, 4 patients did not participate in assessments after chemotherapy cessation during the follow-up (2 of the patients dropped out from the study at this stage and did not complete the study). Among the 75 patients, 63 were assessed 1 year after chemotherapy cessation.

The mean age of the study patients was 57.2 ± 9 years. Among the patients, 64.0% had body mass index (BMI) ≥ 25 kg/m^2^ and 30.7% were obese. The mean BMI was 27.3 ± 5 kg/m^2^. The mean cardiovascular risk calculated using the SCORE chart was 4.9 ± 3. Among comorbidities, hypertension was present in 42 patients (56%), diabetes mellitus in 9 (12%), and coronary artery disease in 3 (4%). There were 31 patients (41.9%) who either were current smokers or had smoked within 5 years prior to enrolment in the study. Baseline assessment revealed dyslipidemia in 51 patients (68.0%).

The most common concomitant medications in the patients were β-blockers (26.8%), followed by angiotensin converting enzyme inhibitors (21.3%), diuretics (21.1%), angiotensin II receptor blockers (sartans) (19.7%), dihydropyridine calcium channel blockers (14.1%), hydroxymethylglutaryl-coenzyme A reductase inhibitors (statins) (13.9%), sulfonylureas (6.7%), biguanides (5.3%), insulin (4.0%), and alpha-glucosidase inhibitors (1.3%).

### Cardiotoxicity

Cardiotoxicity was observed during the follow-up in 14 patients (18.7%). The mean duration of follow-up was 444 ± 101 days, and the median follow-up was 464 ± 64 days. Cardiotoxicity was observed after a mean of 327 ± 148 days from baseline. The minimum and maximum follow-up durations since the last cycle of drug infusion were 78 and 631 days, respectively.

Initially, the echocardiographic parameters, comorbidities, and concomitant medications did not differ between patients with cardiotoxicity and those without cardiotoxicity during the follow-up period. Table 1 presents a complete summary of echocardiographic variables before anthracycline treatment in each of the subgroups.

**Table 1:** Comparison of echocardiography parameters before anthracycline treatment between the subgroups stratified according to cardiotoxicity during follow-up

Characteristic	Without cardiotoxicity (*n* = 61) Mean ± SD (median)	With cardiotoxicity (*n* = 14) Mean ± SD (median)	*p*-value
**EF (%)**	**62.1 ± 5 (62)**	**61.9 ± 6 (60)**	**0.43**
**GLS (%)**	**−20.2 ± 2 (−20.1)**	**−20.6 ± 2 (−20.3)**	**0.89**
IVSd (mm)	9.6 ± 2 (9)	9.8 ± 2 (10)	0.68
IVSs (mm)	12.2 ± 2 (12)	12.3 ± 3 (12)	0.62
LVIDd (mm)	45.6 ± 5 (46)	47.7 ± 3 (47)	0.12
LVIDs (mm)	27.4 ± 4 (27)	29.7 ± 7 (28)	0.36
LVPWd (mm)	10.1 ± 2 (10)	10.3 ± 1 (10)	0.45
LVPWs (mm)	13.5 ± 3 (13)	15.3 ± 3 (15)	0.05
Ao (mm)	28.6 ± 3 (29)	30.2 ± 3 (30.5)	0.06
LA (mm)	37.4 ± 5 (36)	37.7 ± 5 (37)	0.86
Vp (cm/s)	54 ± 20 (48)	50.1 ± 11 (49)	0.85
E wave (m/s)	0.73 ± 0.19 (0.70)	0.73 ± 0.16 (0.75)	0.61
A wave (m/s)	0.81 ± 0.17 (0.82)	0.75 ± 0.20 (0.73)	0.24
E/A	0.92 ± 0.29 (0.85)	1.04 ± 0.37 (0.98)	0.15
IVRT (ms)	104 ± 19 (104)	103 ± 17 (99)	0.81
Tei index	0.467 ± 0.11 (0.469)	0.499 ± 0.12 (0.535)	0.38
E′ med. (cm/s)	8.5 ± 3 (8)	8.7 ± 3 (8)	0.81
A′ med. (cm/s)	10.6 ± 2 (11)	9.5 ± 2 (9)	0.12
S′ med. (cm/s)	6.8 ± 2 (6)	6.9 ± 1 (7)	0.52
E′ lat. (cm/s)	10.3 ± 4 (10)	11.6 ± 2 (11)	0.15
A′ lat. (cm/s)	10.2 ± 3 (10)	10.1 ± 3 (10)	0.83
S′ lat. (cm/s)	7.0 ± 2 (6.5)	6.9 ± 1 (7)	0.41
E′/A′ med.	0.84 ± 0.38 (0.77)	1.02 ± 0.49 (0.74)	0.18
E′/A′ lat.	1.11 ± 0.62 (0.91)	1.24 ± 0.43 (1.12)	0.17

Ao, aorta; EF, ejection fraction; GLS, global longitudinal strain; IVRT, isovolumetric relaxation time; IVSd, intraventricular diastolic diameter; IVSs, intraventricular systolic diameter; LA, left atrium; LVIDd, end-diastolic left ventricular diameter; LVIDs, end-systolic left ventricular diameter; LVPWd, end-diastolic posterior wall diameter; LVPWs, end-systolic posterior wall diameter; Vp, velocity propagation.

No differences in BMI and age were observed between patients with cardiotoxicity and those without cardiotoxicity (median BMI, 25.5 ± 9 kg/m^2^ vs. 26.1 ± 6 kg/m^2^, *p* = 0.82 and median age, 58 ± 8 years vs. 59 ± 8 years, *p* = 0.36). Additionally, there were no differences in anthracycline doses (252 ± 67 mg/m^2^ vs. 245 ± 61 mg/m^2^, *p* = 0.75). Table 2 provides a complete summary of comorbidities and concomitant medications before anthracycline treatment in each of the subgroups.

**Table 2:** Comparison of concomitant medications and comorbidities before anthracycline treatment between the subgroups stratified according to cardiotoxicity during follow-up

Characteristic	Without cardiotoxicity*n* (%)	With cardiotoxicity*n* (%)	*p*-value (Fisher's exact test)
Diabetes	6 (9.8%)	3 (21.4%)	0.35
CAD	3 (4.9%)	0 (0.0%)	0.41
Hypertension	35 (57.4%)	7 (50.0%)	0.77
SCORE risk (mean ± SD) (median)	5.2 ± 3.0 (6)	3.4 ± 2.4 (5)	0.04
Drugs			
β-blockers	16 (28.1%)	3 (21.4%)	0.75
ACE-I	12 (19.7%)	4 (28.6%)	0.48
Diuretics	11 (19.3%)	4 (28.6%)	0.48
Ca-blockers	10 (17.5%)	0 (0.0%)	0.19
Sartans	13 (22.8%)	1 (7.1%)	0.27
Sulfonylureas	3 (4.9%)	2 (14.3%)	0.23
Biguanides	3 (4.9%)	1 (7.1%)	0.57
Insulin	2 (3.3%)	1 (7.1%)	0.47
Dexamethasone	15 (24.6%)	5 (35.7%)	0.52
Smoking	27 (45.0%)	4 (28.6%)	0.37

ACE-I, angiotensin-converting-enzyme inhibitor; Ca, calcium; CAD, coronary artery disease; SCORE, systemic coronary risk evaluation.

**Figure 1:**
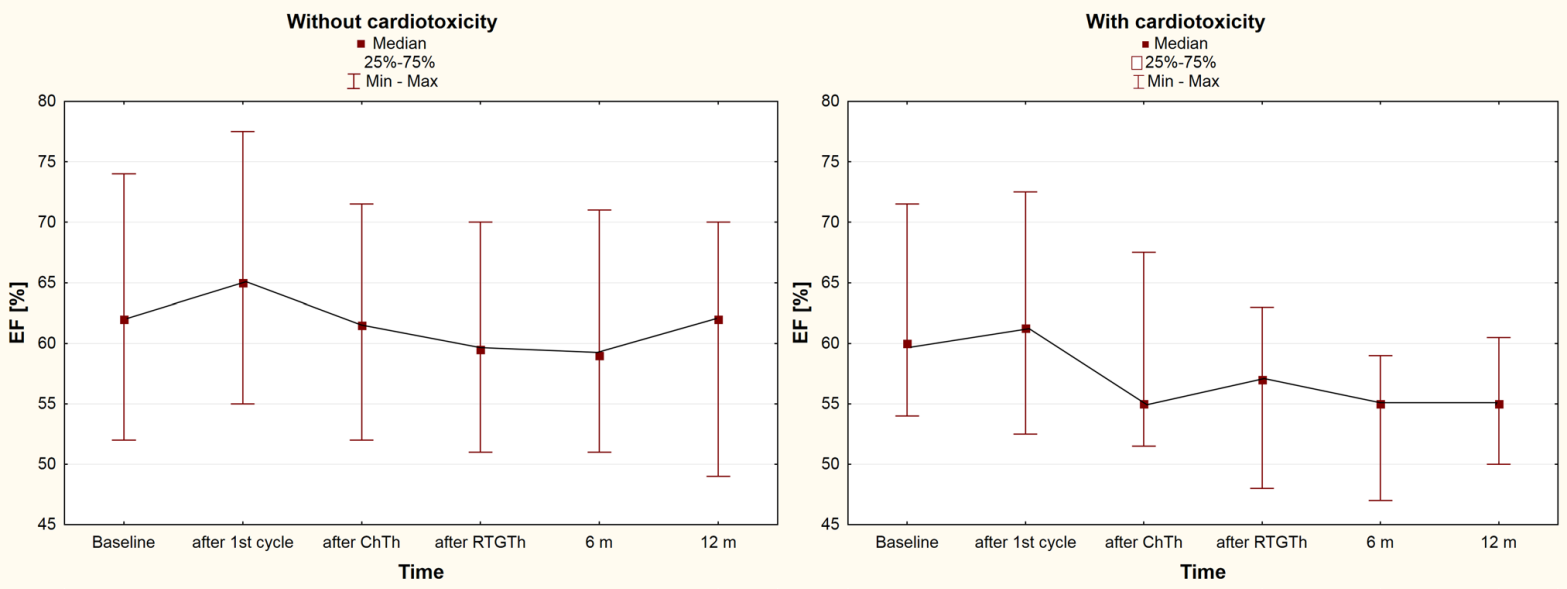
LVEF alterations in the subgroups during the follow-up

During follow-up, after the first cycle of chemotherapy, significant changes were observed in some of the echocardiographic parameters. Improvement in LVEF and longitudinal strain were observed at 24 hours after administration of the cytotoxic agent in the subgroup of patients who did not develop cardiotoxicity in the remainder of the follow-up period. On the other hand, in the subgroup of patients diagnosed with cardiotoxicity during the follow-up, no significant changes in these two key parameters were observed after the first cycle of chemotherapy. Additionally, when we excluded patients who received hypotensive drugs, the improvement was still significant for GLS (subgroups with and without further cardiotoxicity, −19.8 ± 3.6% vs. −21.5 ± 2.8%, respectively). The changes in the subgroup of patients without further cardiotoxicity were transient, and the assessment of LVEF after completion of chemotherapy revealed similar values to those before the treatment. In the subgroup of patients diagnosed with cardiotoxicity, usually later in the study, LVEF significantly declined during the follow-up (Figure 1).

Assessment of diastolic function parameters revealed an improvement in both subgroups. Table 3 provides a complete comparison of the echocardiographic parameters, and Table 4 provides a comparison of the echocardiographic parameters between subgroups after the first cycle of oncological treatment.

**Table 3:** Changes in echocardiographic parameters after the first cycle of chemotherapy

Characteristic	The entire group				Without cardiotoxicity			With cardiotoxicity		
	*n*	Before treatment mean ± SD (median)	After the first cycle of chemotherapy mean ±SD (median)	*p*-value	Before treatment mean ± SD (median)	After the first cycle of chemotherapy mean ± SD (median)	*P*-value	Before treatment mean ± SD (median)	After the first cycle of chemotherapy mean ± SD (median)	*p*-value
**EF (%)**	**75**	**62.1 ± 5 (62)**	**63.8 ± 5 (64)**	**0.01**	**62.1 ± 5 (62)**	64.3 ± 5 (65)	< 0.01	61.9 ± 6 (60)	61.6 ± 7 (61)	0.55
**GLS (%)**	**41**	**−20.1 ± 2 (−20.2)**	**−21.8 ± 2 (−21.8)**	**< 0.01**	**−20.2 ± 2 (−20.1)**	−22.1 ± 2 (−22.0)	< 0.01	−20.6 ± 2 (−20.3)	−20.4 ± 2 (−20.7)	0.16
Vp (cm/s)	69	53 ± 18 (48)	66 ± 25 (62)	**< 0.01**	54 ± 20 (48)	67 ± 26 (62)	**< 0.01**	50 ± 11 (49)	62 ± 24 (66)	0.06
E/A	74	0.95 ± 0.3 (0.87)	1.06 ± 0.3 (0.97)	**< 0.01**	0.92 ± 0.3 (0.85)	1.04 ± 0.3 (0.95)	**0.04**	1.04 ± 0.4 (0.98)	1.14 ± 0.4 (1.05)	**< 0.01**
E′/A′ med.	71	0.87 ± 0.4 (0.77)	0.98 ± 0.4 (0.90)	**< 0.01**	0.84 ± 0.4 (0.77)	0.98 ±0.4 (0.90)	0.19	1.02 ± 0.5 (0.74)	1.00 ± 0.4 (0.88)	**< 0.01**
E′/A′ lat.	70	1.13 ± 0.6 (0.93)	1.24 ± 0.5 (1.13)	**< 0.01**	1.11 ± 0.6 (0.9)	1.28 ± 0.5 (1.16)	0.46	1.23 ± 0.4 (1.11)	1.08 ±0.4 (1.03)	**< 0.01**
IVRT (ms)	72	104 ± 18 (104)	91 ± 19 (90)	**< 0.01**	104 ± 19 (104)	90 ± 18 (89)	**< 0.01**	104 ± 17 (99)	94 ± 20 (93)	0.09
Tei	68	0.473 ± 0.11 (0.472)	0.437 ± 0.12 (0.412)	**< 0.01**	0.468 ± 0.11 (0.469)	0.435 ± 0.12 (0.405)	**0.02**	0.499 ± 0.12 (0.535)	0.446 ± 0.14 (0.414)	**0.04**
E/E′	72	9.0 ± 2 (8.9)	9.2 ± 3 (9.1)	0.81	9.1 ± 2 (8.7)	9.2 ± 3 (8.9)	0.97	9.0 ± 3 (9)	9.3 ± 3 (9.3)	0.79
Ca2+ (mmol/L)	66	2.45 ± 0.1 (2.48)	2.42 ± 0.1 (2.43)	0.11	2.46 ± 0.1 (2.48)	2.42 ± 0.1 (2.43)	**0.03**	2.41 ± 0.1 (2.45)	2.45 ± 0.1 (2.41)	0.27
HR (beats/min)	58	73 ± 10 (73)	73 ± 12 (70)	0.31	74 ± 10 (76)	72 ± 11 (70)	0.19	69 ± 9 (70)	75 ± 16 (76)	0.72

EF, ejection fraction; GLS, global longitudinal strain; HR, heart rate; IVRT, isovolumetric relaxation time; NT-proBNP, N-terminal pro-brain type natriuretic peptide; Vp, velocity propagation.

**Table 4:** Comparison of echocardiography parameters and laboratory test results 24 hours after anthracycline infusion between the subgroups stratified according to cardiotoxicity during follow-up

Characteristic	Without cardiotoxicity (*n* = 61) Mean ± SD (median)	With cardiotoxicity (*n* = 14) Mean ± SD (median)	*p*-value
EF (%)	64.3 ± 5 (65)	61.6 ± 7 (61)	0.16
**GLS (%)**	**−22.1 ± 2 (−22.0)**	**−20.4 ± 2 (−20.7)**	**0.02**
Vp (cm/s)	67 ± 26 (62)	62 ± 24 (66)	0.63
E/A	1.04 ± 0.3 (0.95)	1.14 ± 0.4 (1.05)	0.48
IVRT (ms)	90 ± 18 (89)	94 ± 20 (93)	0.44
Tei index	0.435 ± 0.12 (0.405)	0.446 ± 0.14 (0.414)	0.53
E′ med. (cm/s)	9.6 ± 2.5 (9)	8.5 ± 3.2 (9)	0.39
A′ med. (cm/s)	10.2 ± 2.3 (10)	9.3 ± 1.8 (9)	0.17
S′ med. (cm/s)	7.5 ± 1.6 (8)	7.2 ± 1.6 (8)	0.59
E′ lat. (cm/s)	10.6 ± 3.0 (11)	10.4 ± 2.5 (11)	0.71
A′ lat. (cm/s)	9.8 ± 3.0 (10)	10.4 ± 2.4 (10)	0.46
S′ lat. (cm/s)	7.6 ± 1.8 (7)	8.0 ± 2.4	0.66
E′/A′ med.	0.98 ± 0.38 (0.90)	1.00 ± 0.42 (0.88)	0.95
E′/A′ lat.	1.28 ± 0.54 (1.16)	1.08 ± 0.41 (1.03)	0.22
NT-proBNP (pg/mL)	255 ± 171 (239)	236 ± 138 (241)	0.91

EF, ejection fraction; GLS, global longitudinal strain; IVRT, isovolumetric relaxation time; NT-proBNP, N-terminal pro-brain-type natriuretic peptide; Vp, velocity propagation.

Interestingly, after the first cycle of chemotherapy, no significant differences in echocardiographic parameters were observed between the subgroups, with the exception of GLS, which was significantly worse in the subgroup of patients who developed cardiotoxicity than in the subgroup of patients who did not develop cardiotoxicity (−20.4% vs. −22.1%, *p* = 0.023).

We determined the levels of NT-proBNP and troponin I. The NT-proBNP level significantly increased in both the subgroups 24 hours after anthracycline infusion. There were no differences in the NT-proBNP level between the subgroups. Additionally, the troponin I level was similar between before and 24 hours after anthracycline infusion in all the patients (< 0.01 μg/L).

## DISCUSSION

There are two types of cardiotoxicity. Type I is associated with potentially irreversible myocardial damage, commonly observed in cases of breast cancer with anthracyclines treatment; whereas type II is primarily reversible cardiotoxicity induced by trastuzumab [10, 11]. However, type II can trigger irreversible cardiac damage in patients with existing heart disease or trastuzumab might potentiate anthracyclines cardiotoxicity [11]. Apart from anthracyclines patients in this study also received other oncological drugs such as: doxorubicin (84.0% of the patients) or epirubicin (16.0% of the patients). The mean anthracycline dose, expressed as doxorubicin dose, was similar to other studies and administrated in 4 to 6 cycles. It should be noted that during this study none of the patients who received epirubicin developed cardiotoxicity. This corresponds to larger meta-analysis where epirubicin versus doxorubicin had lower incidence of cardiac events [12]. Cyclophosphamide cardiotoxicity is relatively rare and seen mostly in patients receiving high doses of this drug, usually not given in breast cancer [13]. In our study 82.7% of patients received cyclophosphamide with the mean dose of 61.3 mg/kg. Among other drugs administrated in this group fluorouracil might have induced myocardial ischemia rather than heart failure [14]. Symptoms of ischemia were not observed during this study. Docetaxel was the last chemotherapeutic drug used. Reports about docetaxel's influence on cardiotoxicity are inconclusive. According to some research, docetaxel appears to increase the rate of heart failure [15], but also some reports suggest that taxanes are safer in patients with left ventricle dysfunction [16]. During this observation the difference in cardiotoxicity incidence was not observed among patients, regardless of presence or absence of docetaxel in their treatment.

This research was focused on early alterations in echocardiographic parameters in breast cancer patients. To our knowledge, this is the first study to simultaneously use multiple echocardiographic techniques to assess changes in the myocardium as early as 24 hours after completion of anthracycline infusion. Previous studies, in which the subjects were assessed directly after infusion, focused on changes in laboratory parameters, particularly troponin [17].

Relatively few studies have reported on the overall changes in the myocardium as early as 24 hours after anthracycline administration. One of the first such studies was conducted in 13 patients and involved performing endomyocardial biopsy at the following three time points: before treatment, and 4 and 24 hours after infusion [18]. This previous study demonstrated depression in mitochondrial respiratory activity. Additionally, this previous study performed frequent echocardiographic measurements within the first 24 hours following administration of the cytotoxic agent and demonstrated an improvement in left ventricular systolic function. Subsequent studies demonstrated that most cellular changes (improvement in systolic function) are reversed 24 hours after infusion and that most cells return to the pre-treatment condition [19].

Our study also revealed improvements in both systolic and diastolic function of the left ventricle. However, the improvements were mainly due to the improvement in LVEF in the subgroup of patients who did not subsequently develop cardiotoxicity. A significant improvement was also observed in global longitudinal strain; however, it was only noted in the subgroup without subsequent cardiovascular complications. Although a slight nominal improvement was also found in the other subgroup, the difference was not statistically significant. This might have resulted from the small number of patients in the subgroup with cardiotoxicity. The European Association of Cardiovascular Imaging recommends cardiology consultation when GLS is lower than the normal limit or when the relative percentage reduction in GLS is > 15% [20]. However, in relation to our study, this recommendation would be difficult to implement owing to the fact that at an early stage, only 1 patient had a reduction in GLS of > 15%. Nonetheless, no previous study has assessed GLS at such an early stage of cancer treatment.

It is important to note that in patients undergoing chemotherapy in association with docetaxel, high doses of corticosteroids were administered, which may improve cardiac function [21]. However, in our population, there were no significant differences between the subgroup receiving dexamethasone and the subgroup not receiving dexamethasone as part of the treatment protocol.

Several conflicting reports have been published on the effects of anthracyclines on cardiac myocytes assessed directly after dosing. Some studies in animal models report a positive inotropic effect [22, 23], while others reported a negative inotropic effect [24, 25]. Our study appears to indicate positive inotropism. With regard to diastolic function assessment, beneficial changes were observed, and they were more pronounced and affected a larger number of parameters in the subgroup without subsequent cardiotoxicity than in the subgroup with cardiotoxicity. This might have resulted from a larger number of patients in the subgroup without subsequent cardiotoxicity. A positive inotropic effect in the absence of changes in heart rate suggests a mechanism involving calcium ions [26]. As heart rate was similar in both subgroups, the inotropic effect cannot be explained by conditions, such as a slower heart rate and the action of catecholamines. This is consistent with the findings reported by other authors, who demonstrated increased cytoplasmic levels of calcium ions, resulting from the opening of calcium channels of ryanodine receptors and the release of calcium from the sarcoplasmic reticulum [27, 28]. Anthracyclines have also been shown to inhibit the sodium/calcium exchanger (NCX) and activate L-type calcium channels, thereby increasing the cytoplasmic levels of calcium [26–29]. All these effects result in calcium overload in the cardiac myocytes, increasing their contractile force.

**Figure 2:**
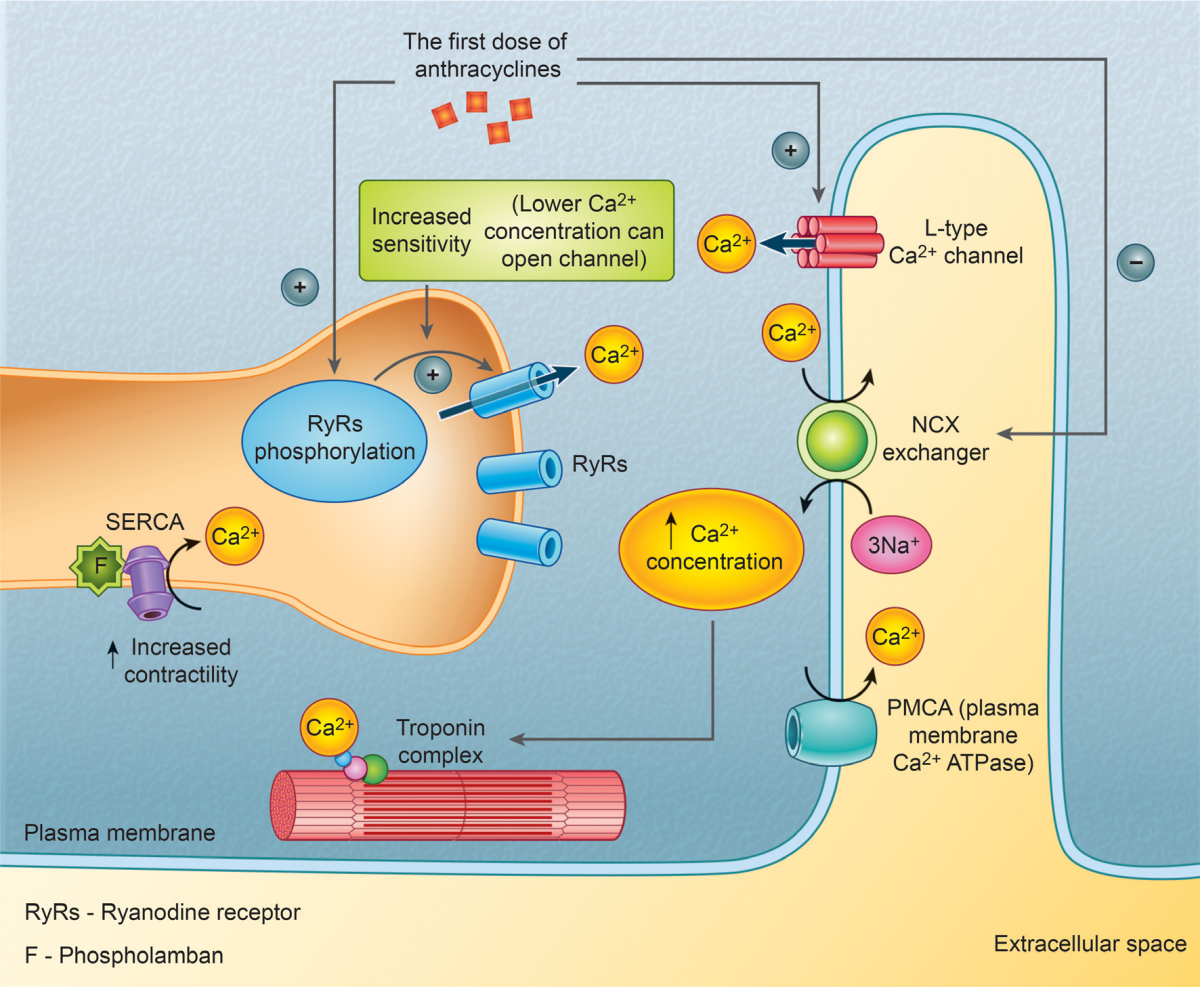
The first dose of anthracyclines’ influence on calcium channels

In the subgroup that did not develop cardiotoxicity during the follow-up, a significant decrease from baseline in the calcium concentration was observed after a single cycle of chemotherapy. This decrease might have resulted from the translocation of calcium ions into the cells as a result of the opening of L-type calcium channels and inhibition of the NCX, which has been confirmed in a recent study [30]. However, no such change in the concentration of calcium ions in the body was observed in the other subgroup. A previous study in an animal model reported an increase in the diffusion of calcium ions from the extracellular space into cardiac myocytes during administration of doxorubicin [31]. On the other hand, in another study, administration of multiple doses of chemotherapy caused desensitization of calcium channels and resulted in a decrease in the calcium ion concentration [32], which would explain the effects observed after completion of chemotherapy in our study, i.e., a return of contractility to pre-treatment levels in the subgroup without cardiotoxicity and a further decline in systolic function in the other subgroup. Other authors have also confirmed that repeated dosing of doxorubicin eventually decreases calcium release from the sarcoplasmic reticulum, which explains the transient nature of the improvement in contractility after the first cycle of chemotherapy in our study [33]. The first dose of anthracyclines’ influence on calcium channels is presented in Figure 2.

In our study, patients who did not demonstrate improvement of left ventricle function were more likely to be diabetic; however, coronary artery disease was not frequently diagnosed in this subgroup. Both risk factors were not statistically significant for predicting cardiotoxicity in this study. Interestingly, the SCORE risk was significantly low in the group with further cardiotoxicity. Therefore, we could not use standard risk factors to predict further left ventricle function impairment. Patients’ age also did not differ between groups with and without further cardiotoxicity. The whole group of patients in this study were women, hence it is difficult to relate potential results of this research to gender.

This study suggests that stimulation of the healthy myocardium with anthracyclines causes an increase of the calcium level in myocardial cells and eventually a good inotropic response, while in hearts that are more prone to cardiotoxicity, this response is not possible with a lower inotropic reserve [34]. Similar situation can be observed in pathophysiology of heart failure. Lower activation efficiency of L-type calcium channel and higher expression of NCX channel are observed in patients with heart failure, hence it is not possible to accumulate higher concentration of calcium ions in smooth endoplasmatic reticulum. According to these findings, cancer sur*vivo*rs with a good inotropic response 24 hours after anthracycline infusion are considered to have a low probability of cardiac events [35, 36].

Our study demonstrated improvements in diastolic function parameters mainly in the subgroup without subsequent cardiotoxicity. In the literature, we did not identified similar measurements performed at such an early period, i.e., no studies assessing subjects 24 hours after administration of chemotherapy have been published. Hence, there are gaps in the scientific information, although it is established that the contraction and relaxation of cardiac myocytes are associated with calcium ions and their movement through ion channels, as described above.

In the present study, we noted that the troponin I level was within the normal range in all patients. However, this finding is in contrast to the finding of a previous study [7]. This difference might be attributable to the fact that we did not assess high-sensitivity troponin [37]. On the other hand, the NT-proBNP level significantly increased just after the first cycle of chemotherapy in the subgroup that developed cardiotoxicity and the subgroup that did not develop this condition; however, there was no difference between the subgroups. Natriuretic peptides have been reported to be closely correlated with heart failure, even in oncological patients [38, 39]. In our study, the level of this marker was significantly high 24 hours after anthracycline infusion; however, the median value was only two times higher than the normal value and was far from the value observed in cases of heart failure. Some papers have mentioned the protective role of β-adrenergic receptors against cardiotoxicity. A previous study in an animal model showed a significantly higher mortality rate in mice deprived of the β-adrenergic receptor than in mice with this receptor after administration of doxorubicin [40]. Additionally, this previous study indicated that the effect was caused by down-regulation of the expression of pro-survival kinases, such as εPKC. We did not observe any changes in heart rate in the study population after one cycle of chemotherapy. Such changes could occur if the number of β-adrenergic receptors changes. Additionally, the percentage of patients taking β-adrenergic receptor blockers in each of the two subgroups was similar. It is therefore impossible to definitely conclude that this is the underlying mechanism of the very early changes in patients after a single cycle of chemotherapy. Moreover, when we excluded patients who received hypotensive drugs, the effect of contractility improvement persisted and remained statistically significant.

This study presents a mechanism in which the first dose of anthracyclines affects cardiac cells. It helps clinicians predict probable cardiac complications in a group of patients, especially those without a good inotropic response at a very early stage – 24 hours after the first infusion of anthracyclines.

Thus, this research suggests the necessity of additional echocardiography exam in the mentioned time and paying special attention to global longitudinal strain assessed by means of speckle tracking. Longitudinal strain measures after the first cycle of drug infusion has a special value for clinicians, for an increase of more than 10% from baseline significantly decreases the risk of further cardiotoxicity. Ejection fraction at this stage has lower usefulness, because mean increase was just over 2 percentage points. On the other hand, the lack of improvement in GLS at the very early stage helps clinicians find a group of patients more liable to cardiac complications who need further thorough observation. Hence global longitudinal strain assessed by means of speckle tracking 24 hours after the first dose of anthracycline is a good parameter for clinicians, allowing to predict further cardiac complications and, on the other hand, making it easier to find a group with lower risk of such incidences.

### Limitations

The present study had some limitations. The number of patients in the subgroups was limited, especially in the subgroup with cardiotoxicity. For confirmation of our results, further studies with a larger population should be performed.

## MATERIALS AND METHODS

This prospective observational clinical study included 75 chemotherapy-naive female patients without heart failure, who were diagnosed with breast cancer and were scheduled to receive anthracycline-based chemotherapy. The exclusion criteria were as follows: atrial fibrillation, previous chemotherapy or radiotherapy irrespective of indication, age > 75 years, valvular regurgitation or stenosis that was more severe than a mild condition, an implanted artificial cardiac valve, and an implanted cardiac pacemaker. Each patient provided written informed consent to participate in the study, and the study was performed in accordance with the Declaration of Helsinki.

During their visits to the Heart Center, the patients underwent echocardiography. The schedule of echocardiographic monitoring was as follows: before treatment (up to 7 days before dosing), 24 hours after completion of the first cycle of chemotherapy, 24 hours after chemotherapy cessation, at the end of radiotherapy, and 6 and 12 months after chemotherapy cessation.

Echocardiography was performed by an experienced echocardiographer using Vivid E9 (GE Medical Systems, Milwaukee, WI) and the 4V-3D transducer (1.5–4.0 MHz; GE Medical Systems). At each stage of the study, patients underwent medical examination and were asked detailed questions about symptoms, particularly symptoms indicative of heart failure.

Full transthoracic echocardiography was performed in the left lateral decubitus position. The images were acquired in the parasternal, apical four-chamber, and substernal views. LVEF was assessed in the two- and four-chamber views with the Simpson method, as recommended by the European Society of Echocardiography [41]. The measurements were performed in real time, without reference to previous results. The cardiotoxicity endpoint was defined as a decline in the ejection fraction (EF) of ≥ 5% to < 55% accompanied by clinical manifestations of heart failure assessed at the Heart Center or a decline in the EF of ≥ 10% to < 55% unaccompanied by clinical manifestations of heart failure [6].

Left ventricular end-diastolic and end-systolic diameters, interventricular septum thickness in diastole and systole, and posterior wall thickness in diastole and systole were assessed in the parasternal long-axis view just below the mitral valve leaflets. The left atrial anteroposterior diameter was measured in early diastole, and the aortic diameter was measured in late diastole.

Mitral inflow velocity was recorded in the apical four-chamber view at the tip of the mitral valve leaflets using pulsed-wave Doppler (recording of E-wave deceleration time and A-wave velocity). Mitral inflow propagation velocity (Vp) was also measured in the four-chamber view using color Doppler in M-mode along the central portion of the mitral annulus. Vp defined the slope of the signal contour line. The apical five-chamber view was used to measure isovolumetric relaxation time (IVRT), mitral valve closure time (MCOT), and left ventricular ejection time (LVET). For these assessments, the continuous-wave Doppler sample gate was placed at the anterior leaflet of the mitral valve to record left ventricular outflow, approximately halfway between the mitral and aortic valves. The Tei index was calculated automatically by the device using the following formula: Tei index = (MCOT − LVET)/LVET, where MCOT signified time from mitral valve closure to opening. Tissue Doppler was used in the four-chamber view to assess E′-, A′-, and S′-wave velocities in both the medial and lateral positions. The Doppler sample gate was placed approximately 1 cm towards the apex from the medial and lateral portions of the mitral annulus each time. For all the Doppler measurements, the velocity of scanning was adjusted individually to achieve maximum accuracy of the measurements (filling the screen with the cardiac cycle).

The scanning depth and diagnostic window width were adjusted such that the left ventricle filled the window to the fullest extent possible, and two-,three-, and four-chamber views were recorded. The frame rate ranged between 50 and 80 frames per second. Based on the views recorded at examination (with the patient present), global longitudinal strain (GLS) was analyzed using an application included in the device for the assessment of speckle tracking strain. Left ventricular systole was defined as the interval between aortic valve opening and closure. During the examination, electrocardiography (ECG) tracing (one lead) was simultaneously performed. Regions used for the analysis of left ventricular global longitudinal strain were selected manually. The regions were selected by first marking the endocardial border in two basal segments and in the apex of the views in late diastole and then manually correcting the tracking speckles from each of the segments to confirm the correct marking of each of the regions. At this point, if necessary, the thickness of the marked regions was corrected using the included application (region of interest). If two or less unacceptable segments remained, the echocardiographer considered the quality of the recording sufficient, and the speckle tracking reflected the actual movements of the myocardium, the examination was approved and the GLS assessment was considered complete. Images with more than two unacceptable segments due to poor image quality were rejected from the final analysis, and for such an examination, the GLS value was neither provided nor included in further analysis. Global longitudinal strain was calculated after the final approval of segments from the three views mentioned above. In the final step, the application included in the device calculated the mean value for all 18 segments.

Laboratory tests were performed. N-terminal pro-brain-type natriuretic peptide (NT-proBNP) levels were assessed using the electrochemiluminescence method and troponin I (standard troponin I and not high-sensitivity troponin I) levels were assessed using immunochemical techniques.

### Administration of anthracyclines

Anthracyclines were administered at the Oncology Center. Doses of anthracyclines (doxorubicin and epirubicin) were calculated from the dose levels expressed in mg/m^2^ using the Cato^®^ system (Quality Management for Oncology Therapy). For the purpose of statistical calculation, in order to unify anthracycline doses, we used the following commonly available formula to convert epirubicin doses: epirubicin dose × 0.6 = doxorubicin dose equivalent [42].

### Statistics

The study variables are expressed as means and standard deviations (SD) or medians and interquartile ranges (IQR), as appropriate. Changes during treatment were assessed and subsequently analyzed using the Wilcoxon signed-rank test. To compare patients who developed cardiotoxicity with those who did not develop cardiotoxicity, the Mann-Whitney *U* test was used for quantitative variables and the chi-square test with Fisher's correction for nominal values was used for qualitative variables. Statistical calculations were performed using the Statistica 12 software (StatSoft Inc., Tulsa, OK). A *p*-value < 0.05 was considered statistically significant.

## CONCLUSIONS

In conclusion, to our knowledge, this study is the first research that demonstrated echocardiographic changes occurring within 24 hours after administration of chemotherapy. The findings of our study fill the gap in information on the early stage of oncological treatment. Additionally, our findings suggest that transient improvement in contractility and systolic and diastolic function might occur 24 hours after anthracycline administration, especially in patients who do not develop cardiotoxicity. The observed alterations were independent from concomitant drugs, diseases and patients’ age. Earlier evaluation of patients during chemotherapy is worth considering, because those without a good inotropic response to anthracycline infusion are more prone to develop cardiotoxicity and the improvement of GLS after 1st cycle allows to find a group of patients with lower risk of further cardiotoxicity.

## References

[1] Singal PK, Li T, Kumar D, Danelisen I, Iliskovic N (2000). Adriamycin-induced heart failure: mechanism and modulation. Mol Cell Biochem.

[2] Damiani RM, Moura DJ, Viau CM, Caceres RA, Henriques JA, Saffi J (2016). Pathways of cardiac toxicity: comparison between chemotherapeutic drugs doxorubicin and mitoxantrone. Arch Toxicol.

[3] Curigliano G, Cardinale D, Suter T, Plataniotis G, de Azambuja E, Sandri MT, Criscitiello C, Goldhirsch A, Cipolla C, Roila F, ESMO Guidelines Working Group (2012). Cardiovascular toxicity induced by chemotherapy, targeted agents and radiotherapy: ESMO Clinical Practice Guidelines. Ann Oncol.

[4] Villarraga HR, Herrmann J, Nkomo VT (2014). Cardio-oncology: role of echocardiography. Prog Cardiovasc Dis.

[5] Curtis JP, Sokol SI, Wang Y, Rathore SS, Ko DT, Jadbabaie F, Portnay EL, Marshalko SJ, Radford MJ, Krumholz HM (2003). The association of left ventricular ejection fraction, mortality, and cause of death in stable outpatients with heart failure. J Am Coll Cardiol.

[6] Seidman A, Hudis C, Pierri MK, Shak S, Paton V, Ashby M, Murphy M, Stewart SJ, Keefe D (2002). Cardiac dysfunction in the trastuzumab clinical trials experience. J Clin Oncol.

[7] Sawaya H, Sebag IA, Plana JC, Januzzi JL, Ky B, Tan TC, Cohen V, Banchs J, Carver JR, Wiegers SE, Martin RP, Picard MH, Gerszten RE (2012). Assessment of echocardiography and biomarkers for the extended prediction of cardiotoxicity in patients treated with anthracyclines, taxanes, and trastuzumab. Circ Cardiovasc Imaging.

[8] Cardinale D, Colombo A, Lamantia G, Colombo N, Civelli M, De Giacomi G, Rubino M, Veglia F, Fiorentini C, Cipolla CM (2010). Anthracycline-induced cardiomyopathy: clinical relevance and response to pharmacologic therapy. J Am Coll Cardiol.

[9] McGowan JV, Chung R, Maulik A, Piotrowska I, Walker JM, Yellon DM (2017). Anthracycline Chemotherapy and Cardiotoxicity. Cardiovasc Drugs Ther.

[10] Ewer MS, Vooletich MT, Durand JB, Woods ML, Davis JR, Valero V, Lenihan DJ (2005). Reversibility of trastuzumab-related cardiotoxicity: new insights based on clinical course and response to medical treatment. J Clin Oncol.

[11] Suter TM, Ewer MS (2013). Cancer drugs and the heart: importance and management. Eur Heart J.

[12] Smith LA, Cornelius VR, Plummer CJ, Levitt G, Verrill M, Canney P, Jones A (2010). Cardiotoxicity of anthracycline agents for the treatment of cancer: systematic review and meta-analysis of randomised controlled trials. BMC Cancer.

[13] Zamorano JL, Lancellotti P, Rodriguez Muñoz D, Aboyans V, Asteggiano R, Galderisi M, Habib G, Lenihan DJ, Lip GY, Lyon AR, Lopez Fernandez T, Mohty D, Piepoli MF (2016). 2016 ESC Position Paper on cancer treatments and cardiovascular toxicity developed under the auspices of the ESC Committee for Practice Guidelines: The Task Force for cancer treatments and cardiovascular toxicity of the European Society of Cardiology (ESC). Eur Heart J.

[14] Polk A, Vistisen K, Vaage-Nilsen M, Nielsen DL (2014). A systematic review of the pathophysiology of 5-fluorouracil-induced cardiotoxicity. BMC Pharmacol Toxicol.

[15] Mackey JR, Martin M, Pienkowski T, Rolski J, Guastalla JP, Sami A, Glaspy J, Juhos E, Wardley A, Fornander T, Hainsworth J, Coleman R, Modiano MR, TRIO/BCIRG 001 investigators (2013). TRIO/BCIRG 001 investigators. Adjuvant docetaxel, doxorubicin, and cyclophosphamide in node-positive breast cancer: 10-year follow-up of the phase 3 randomised BCIRG 001 trial. Lancet Oncol.

[16] Gollerkeri A, Harrold L, Rose M, Jain D, Burtness BA (2001). Use of paclitaxel in patients with pre-existing cardiomyopathy: a review of our experience. Int J Cancer.

[17] Stachowiak P, Kornacewicz-Jach Z, Safranow K (2014). Prognostic role of troponin and natriuretic peptides as biomarkers for deterioration of left ventricular ejection fraction after chemotherapy. Arch Med Sci.

[18] Unverferth DV, Magorien RD, Unverferth BP, Talley RL, Balcerzak SP, Baba N (1981). Human myocardial morphologic and functional changes in the first 24 hours after doxorubicin administration. Cancer Treat Rep.

[19] Unverferth BJ, Magorien RD, Balcerzak SP, Leier CV, Unverferth DV (1983). Early changes in human myocardial nuclei after doxorubicin. Cancer.

[20] Plana JC, Galderisi M, Barac A, Ewer MS, Ky B, Scherrer-Crosbie M, Ganame J, Sebag IA, Agler DA, Badano LP, Banchs J, Cardinale D, Carver J (2014). Expert consensus for multimodality imaging evaluation of adult patients during and after cancer therapy: a report from the American Society of Echocardiography and the European Association of Cardiovascular Imaging. Eur Heart J Cardiovasc Imaging.

[21] Ballo P, Motto A, Corsini F, Orlandini F, Mondillo S (2008). Early improvement in cardiac function detected by tissue Doppler and strain imaging after melphalan-dexamethasone therapy in a 51-year old subject with severe cardiac amyloidosis. Int J Cardiol.

[22] Kim DH, Akera T, Brody TM (1980). Inotropic actions of doxorubicin in isolated guinea-pig atria: evidence for lack of involvement of Na+,K+-adenosine triphosphatase. J Pharmacol Exp Ther.

[23] Temma K, Akera T, Chugun A, Ohashi M, Yabuki M, Kondo H (1992). Doxorubicin: an antagonist of muscarinic receptors in guinea pig heart. Eur J Pharmacol.

[24] de Jong J, Schoofs PR, Onderwater RC, van der Vijgh WJ, Pinedo HM, Bast A (1990). Isolated mouse atrium as a model to study anthracycline cardiotoxicity: the role of the beta-adrenoceptor system and reactive oxygen species. Res Commun Chem Pathol Pharmacol.

[25] Höfling B, Bolte HD (1981). Acute negative inotropic effect of adriamycin (doxorubicin). Naunyn Schmiedebergs Arch Pharmacol.

[26] Asensio-Lopez MC, Soler F, Sanchez-Mas J, Pascual-Figal D, Fernandez-Belda F, Lax A (2016). Early oxidative damage induced by doxorubicin: Source of production, protection by GKT137831 and effect on Ca(2+) transporters in HL-1 cardiomyocytes. Arch Biochem Biophys.

[27] Hanna AD, Lam A, Tham S, Dulhunty AF, Beard NA (2014). Adverse effects of doxorubicin and its metabolic product on cardiac RyR2 and SERCA2A. Mol Pharmacol.

[28] Kim SY, Kim SJ, Kim BJ, Rah SY, Chung SM, Im MJ, Kim UH (2006). Doxorubicin-induced reactive oxygen species generation and intracellular Ca2+ increase are reciprocally modulated in rat cardiomyocytes. Exp Mol Med.

[29] Hanna AD, Janczura M, Cho E, Dulhunty AF, Beard NA (2011). Multiple actions of the anthracycline daunorubicin on cardiac ryanodine receptors. Mol Pharmacol.

[30] Tocchetti CG, Carpi A, Coppola C, Quintavalle C, Rea D, Campesan M, Arcari A, Piscopo G, Cipresso C, Monti MG, De Lorenzo C, Arra C, Condorelli G (2014). Ranolazine protects from doxorubicin-induced oxidative stress and cardiac dysfunction. Eur J Heart Fail.

[31] Rinaldi B, Di Pierro P, Vitelli MR, D’Amico M, Berrino L, Rossi F, Filippelli A (2002). Effects of docosahexaenoic acid on calcium pathway in adult rat cardiomyocytes. Life Sci.

[32] Pessah IN (1992). Calcium release channel of sarcoplasmic reticulum: an important target for doxorubicin-mediated cardiotoxicity. Adv Exp Med Biol.

[33] Dodd DA, Atkinson JB, Olson RD, Buck S, Cusack BJ, Fleischer S, Boucek RJ (1993). Doxorubicin cardiomyopathy is associated with a decrease in calcium release channel of the sarcoplasmic reticulum in a chronic rabbit model. J Clin Invest.

[34] Senior R, Monaghan M, Becher H, Mayet J, Nihoyannopoulos P, British Society of Echocardiography (2005). Stress echocardiography for the diagnosis and risk stratification of patients with suspected or known coronary artery disease: a critical appraisal. Heart.

[35] Kirkham AA, Virani SA, Campbell KL (2015). The utility of cardiac stress testing for detection of cardiovascular disease in breast cancer survivors: a systematic review. Int J Womens Health.

[36] Chandra S, Lenihan DJ, Wei W, Yusuf SW, Tong AT (2009). Myocardial perfusion imaging and cardiovascular outcomes in a cancer population. Tex Heart Inst J.

[37] Xue K, Gu JJ, Zhang Q, Liu X, Wang J, Li XQ, Luo J, Hernandez-Ilizaliturri FJ, Fernandez SF, Czuczman MS, Cao J, Hong X, Guo Y (2016). Cardiotoxicity as indicated by LVEF and troponin T sensitivity following two anthracycline-based regimens in lymphoma: Results from a randomized prospective clinical trial. Oncotarget.

[38] Sandri MT, Salvatici M, Cardinale D, Zorzino L, Passerini R, Lentati P, Leon M, Civelli M, Martinelli G, Cipolla CM (2005). N-terminal pro-B-type natriuretic peptide after high-dose chemotherapy: a marker predictive of cardiac dysfunction?. Clin Chem.

[39] Lenneman CG, Sawyer DB (2016). Cardio-Oncology: An Update on Cardiotoxicity of Cancer-Related Treatment. Circ Res.

[40] Fajardo G, Zhao M, Berry G, Wong LJ, Mochly-Rosen D, Bernstein D (2011). β2-adrenergic receptors mediate cardioprotection through crosstalk with mitochondrial cell death pathways. J Mol Cell Cardiol.

[41] Lang RM, Badano LP, Mor-Avi V, Afilalo J, Armstrong A, Ernande L, Flachskampf FA, Foster E, Goldstein SA, Kuznetsova T, Lancellotti P, Muraru D, Picard MH (2015). Recommendations for cardiac chamber quantification by echocardiography in adults: an update from the American Society of Echocardiography and the European Association of Cardiovascular Imaging. Eur Heart J Cardiovasc Imaging.

[42] Cottin Y, Touzery C, Dalloz F, Coudert B, Toubeau M, Riedinger A, Louis P, Wolf JE, Brunotte F (1998). Comparison of epirubicin and doxorubicin cardiotoxicity induced by low doses: evolution of the diastolic and systolic parameters studied by radionuclide angiography. Clin Cardiol.

